# Allele frequencies of single nucleotide polymorphisms of clinically important drug-metabolizing enzymes *CYP2C9*, *CYP2C19*, and *CYP3A4* in a Thai population

**DOI:** 10.1038/s41598-021-90969-y

**Published:** 2021-06-11

**Authors:** Rattanaporn Sukprasong, Sumonrat Chuwongwattana, Napatrupron Koomdee, Thawinee Jantararoungtong, Santirhat Prommas, Pimonpan Jinda, Jiratha Rachanakul, Nutthan Nuntharadthanaphong, Nutcha Jongjitsook, Apichaya Puangpetch, Chonlaphat Sukasem

**Affiliations:** 1 Division of Pharmacogenomics and Personalized Medicine, Department of Pathology, Faculty of Medicine Ramathibodi Hospital, Mahidol University, Bangkok, 10400 Thailand; 2 Laboratory for Pharmacogenomics, Somdech Phra Debaratana Medical Center (SDMC), Ramathibodi Hospital, Bangkok, Thailand; 3 Faculty of Medical Technology, Huachiew Chalermprakiet University, Bang Phli District, Thailand

**Keywords:** Genetics, Medical research, Molecular medicine

## Abstract

Prior knowledge of allele frequencies of cytochrome P450 polymorphisms in a population is crucial for the revision and optimization of existing medication choices and doses. In the current study, the frequency of the CYP2C9*2, CYP2C9*3, CYP2C19*2, CYP2C19*3, CYP2C19*6, CYP2C19*17, and *CYP3A4* (rs4646437) alleles in a Thai population across different regions of Thailand was examined. Tests for polymorphisms of *CYP2C9* and *CYP3A4* were performed using TaqMan SNP genotyping assay and *CYP2C19* was performed using two different methods; TaqMan SNP genotyping assay and Luminex x Tag V3. The blood samples were collected from 1205 unrelated healthy individuals across different regions within Thailand. Polymorphisms of *CYP2C9* and *CYP2C19* were transformed into phenotypes, which included normal metabolizer (NM), intermediate metabolizer (IM), poor metabolizer (PM), and rapid metabolizers (RM). The *CYP2C9* allele frequencies among the Thai population were 0.08% and 5.27% for the CYP2C9*2 and CYP2C9*3 alleles, respectively. The *CYP2C19* allele frequencies among the Thai population were 25.60%, 2.50%, 0.10%, and 1.80% for the CYP2C19*2, CYP2C19*3, CYP2C19*6, and CYP2C19*17 alleles, respectively. The allele frequency of the *CYP3A4* (rs4646437) variant allele was 28.50% in the Thai population. The frequency of the CYP2C9*3 allele was significantly lower among the Northern Thai population (P < 0.001). The frequency of the CYP2C19*17 allele was significantly higher in the Southern Thai population (P < 0.001). Our results may provide an understanding of the ethnic differences in drug responses and support for the utilization of pharmacogenomics testing in clinical practice.

## Introduction

Genetic variations exist across different human populations and are often associated with the variation of drug response between populations^1^. Pharmacogenomics is the study of genetic variants that influence drug effects, typically through alterations in pharmacokinetics and pharmacodynamics^2^. Genetic polymorphisms in phase-1 drug-metabolizing enzymes, such as cytochrome P450 oxidases (CYPs), can alter the pharmacokinetic properties of the administered drugs, their metabolites, or both at the target site, resulting in variability in drug responses^3^. The genetic variability in CYP enzymes results in an enzyme with increased, normal, decreased, or no enzyme activity^3^. CYP enzymes consist of 57 functional members, which are classified into 18 families and 43 subfamilies based on sequence similarity. CYP enzymes metabolize the majority of the common clinically prescribed drugs^4^. Polymorphisms in *CYP* genes categorize the population as poor metabolizer (PM), intermediate metabolizer (IM), normal metabolizer (NM), rapid metabolizer (RM) and ultrarapid metabolizer (UM)^5^. Genetic polymorphisms within *CYP2C9*, *CYP2C19*, and *CYP3A4* significantly affect most clinically used drugs, and the prediction of phenotypes by detecting polymorphisms of these *CYP* genes is useful in drug therapy.

The CYP2C9*2 (R144C) and CYP2C9*3 (I359L) alleles are the clinically relevant defective variants associated with decreased enzyme activity and impaired drug metabolism phenotypes. CYP2C9 metabolizes approximately 25% of clinically-administered drugs including phenytoin, warfarin, glipizide, tolbutamide and non-steroidal anti-inflammatory drugs (NSAIDs)^2,3^. The frequency of CYP2C9*2 is 12.68% in European, 4.60% in South Asian, 2.35% in African, and < 1% in East Asian ancestry. The frequency of CYP2C9*3 is 11.31% in South Asian, 6.88% in European, 3.38% in East Asian, and 1.26% in African ancestry^6^.

Among the *CYP2C19* polymorphisms, CYP2C19*2, CYP2C19*3, CYP2C19*6, and CYP2C19*17 are the common variants responsible for interindividual differences in the pharmacokinetics and response to CYP2C19 substrates^7^. CYP2C19 is involved in the metabolism of tricyclic antidepressants, selective serotonin reuptake inhibitors, voriconazole, and clopidogrel^8^. The allelic frequencies of CYP2C19*2 and CYP2C19*3 responsible for the majority of PM phenotypes in the metabolism of CYP2C19 substrate drugs are higher in Asian populations^9^.

CYP3A4 is responsible for the metabolism of ∼50% of drugs used therapeutically such as clarithromycin, erythromycin, diltiazem, itraconazole, ketoconazole, ritonavir. There is considerable interindividual variability in CYP3A4 activity^10^. *CYP3A4* rs4646437 polymorphism was related to the risk of hypertension in the Chinese population^11^. Assessment of inter-individual differences in the allele and genotype frequencies in the Thai population is important for the better outcome of pharmacotherapy. In this study, we have analyzed the frequency of specific gene polymorphisms of *CYP2C9*, *CYP2C19*, and *CYP3A4* (Table 1) in the Thai population. We also compared the the real-time polymerase chain reaction technique (real-time PCR) (ViiA7) and Luminex bead-based multiplex assays for the detection of *CYP2C19* variants.

**Table 1:** Locations and effects of *CYP2C9*,* CYP2C19*, and *CYP3A4* polymorphisms (adopted from^12,13^).

Polymorphism	Nucleotide change	rs number*	Location, protein effect	Enzyme activity
CYP2C9*2	430C > T	rs1799853	R144C	Decreased
CYP2C9*3	1075A > C	rs1057910	I359L	Decreased
CYP2C19*2	681G > A	rs4244285	Splicing defect	Null allele
CYP2C19*3	636G > A	rs4986893	W212X	Null allele
CYP2C19*6	395G > A	rs72552267	R132Q	Null allele
CYP2C19*17	-806C > T	rs12248560	I331V	Increased
*CYP3A4*	C > T	rs4646437	Intron variant	–

*rs number—reference Single Nucleotide Polymorphism (SNP) ID assigned by the SNP database at National Center for Biotechnology Information (dbSNP).

## Methods

### Subjects

For the allele frequency of *CYP2C9*, *CYP2C19*, and *CYP3A4* polymorphisms, a total of 1,205 blood specimens of unrelated healthy donors were obtained from the Thai National Health Examination Survey. The samples were selected from five regions of Thailand, including Northern, Northeastern, Central, Southern, and Bangkok. For the comparison between the real-time PCR technique (ViiA7) and Luminex bead-based multiplex assays for the detection *CYP2C19* variants, voriconazole-treated patients (N = 180) were recruited between 2012 to 2015 from the Division of Infectious Disease, Department of Medicine, Faculty of Medicine, Ramathibodi Hospital, Mahidol University, Thailand. This study was approved by the ethical committee of the Ramathibodi Hospital, Faculty of Medicine, Mahidol University (MURA2013/292/SP_6_) and conducted in accordance with the Declaration of Helsinki. The study protocol was clearly explained to all participants and/or their legal guardians, and informed consent was obtained before the study.

### Genomic DNA extraction and genotyping

Genomic DNA was extracted from EDTA blood sample using a MagNA Pure LC DNA Isolation Kit I (Roche, Mannheim, Germany) and quantified using NanoDrop ND-1000 Spectrophotometer (Thermo Fisher Scientific, DE, USA). TaqMan SNP Genotyping Assays of CYP2C9*2, CYP2C9*3, CYP2C19*2, CYP2C19*3, CYP2C19*6, CYP2C19*17, and *CYP3A4* (rs4646437) were performed on the ViiA 7 real-time PCR System (Applied Biosystems, Foster City, California), according to the manufacturer's instructions. *CYP2C19* genotyping using Luminex xTAG v3 was performed on the Luminex 100/200 System (Luminex Molecular Diagnostics Inc., Austin, Texas).

### Statistical analysis

Statistical analyses were performed using the SPSS software package (SPSS version 18.0 for Windows, SPSS Inc., Chicago, IL, https://www.ibm.com/products/spss-statistics ). The genotype distributions were evaluated for Hardy–Weinberg equilibrium by using Fisher’s exact test. Allele frequencies in the Thai population among the different regions of Thailand were compared using the χ2-test. P-value < 0.05 was considered significance. Cohen's Kappa (κ) was used to calculate the agreement between the Luminex xTAG v3 and TaqMan SNP genotyping methods.

## Results

### Genotype and allele frequencies of polymorphisms of *CYP2C9*, *CYP2C19*, and *CYP3A4* in Thai

The genotype and allele frequencies for all the polymorphisms screened are shown in Table 2. All the genotypes were in Hardy–Weinberg equilibrium (P > 0.05). The sample size for the analysis was 1,205. The major allele was defined as the most commonly occurring allele in the population. The population allele frequencies were calculated from genotype numbers.

**Table 2:** Genotype and allele frequencies of *CYP2C9*, *CYP2C19*, and *CYP3A4* polymorphism in a Thai population (n = 1205).

Gene	Allele	Genotype	Genotype frequency, n (%)	Minor allele frequency
*CYP2C9*	CYP2C9*2	CC	1203 (99.83)	T = 0.0008
		CT	2 (0.17)	
	CYP2C9*3	AA	1080 (95.19)	C = 0.0527
		AC	123 (4.73)	
		CC	2 (0.08)	
*CYP2C19*	CYP2C19*2	GG	652 (54.11)	A = 0.256
		GA	489 (40.58)	
		AA	64 (5.31)	
	CYP2C19*3	GG	1147 (95.19)	A = 0.025
		GA	57 (4.73)	
		AA	1 (0.08)	
	CYP2C19*6	GG	1203 (99.83)	A = 0.001
		GA	2 (0.17)	
	CYP2C19*17	CC	1162 (96.43)	T = 0.018
		CT	43 (3.57)	
*CYP3A4*	*CYP3A4* (rs4646437)	GG	610 (50.62)	A = 0.285
		GA	506 (41.99)	
		AA	89 (7.39)	

In our sample of Thai unrelated healthy population, two individuals were heterozygous for the CYP2C9*2 variant. CYP2C9*2 variant allele frequency was 0.08%. Carriers of the CYP2C9*3 variant allele were found in 125 individuals; two of these individuals were homozygous for the CYP2C9*3 variant while 123 individuals were heterozygous for the CYP2C9*3 variant. CYP2C9*3 variant allele frequency was 5.27%.

Regarding *CYP2C19* variants, the homozygous CYP2C19*2 variant was found in 64 individuals, while 489 individuals were heterozygous for the CYP2C19*2 variant. The allele frequency of the CYP2C19*2 variant was 25.6%. Fifty-eight individuals carried the CYP2C19*3 variant allele; one individual was homozygous for the CYP2C19*3 variant while 57 individuals were heterozygous for the CYP2C19*3 variant. CYP2C19*3 variant allele frequency was 2.5%. The heterozygous CYP2C19*6 variant was found in two individuals. CYP2C19*6 variant allele frequency was 0.1%. Forty-three individuals were heterozygous for the CYP2C19*17 variant, and the CYP2C19*17 variant allele frequency was 1.8%.

Carriers of the *CYP3A4* (rs4646437) variant allele were found in 595 individuals in this study population; 89 of these individuals were homozygous for the rs4646437 variant while 506 individuals were heterozygous for the rs4646437 variant. The allele frequency of the rs4646437 variant allele was 28.5%.

### Frequency distribution of polymorphisms of *CYP2C9*, *CYP2C19*, and *CYP3A4* in different regions of Thailand

**Figure 1:**
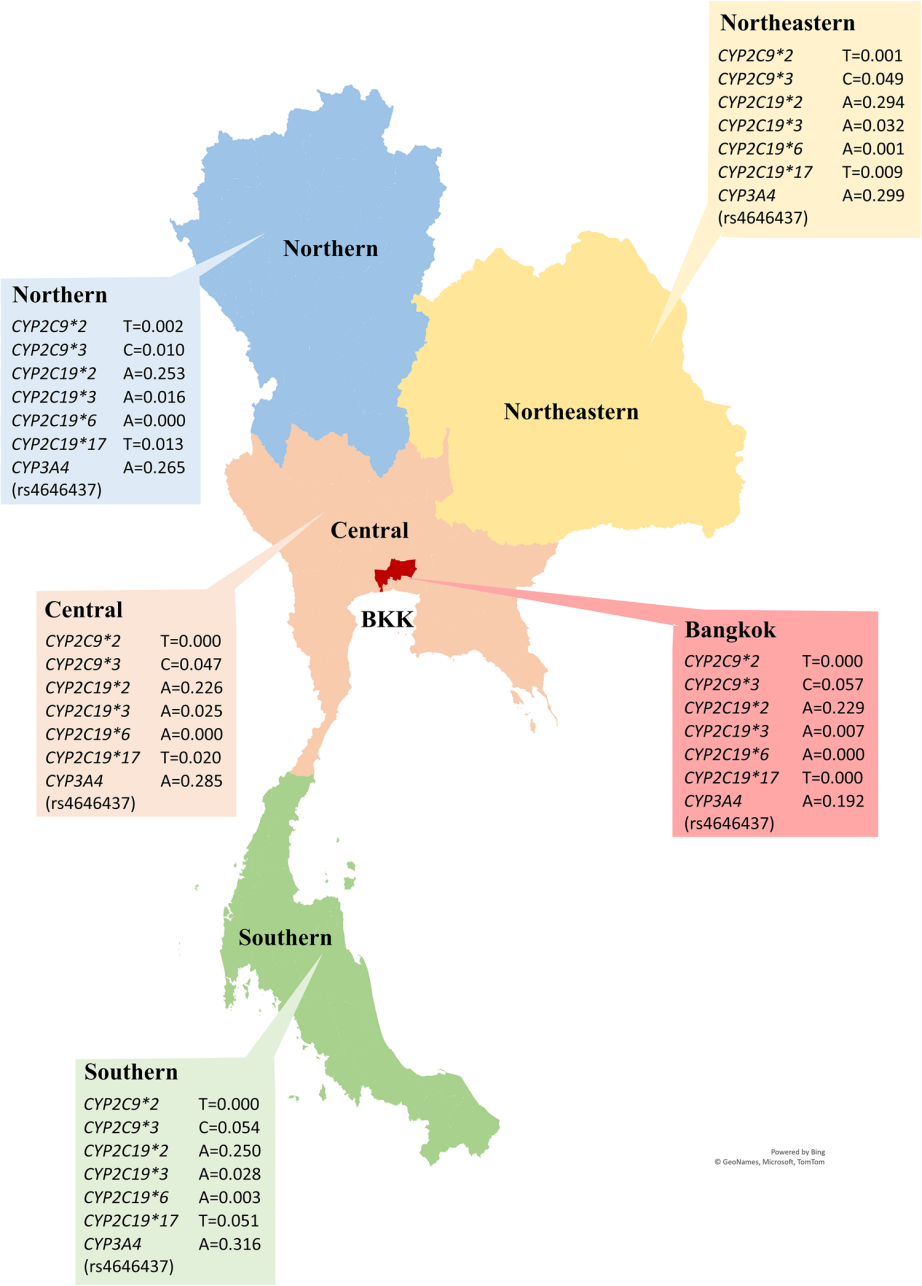
The distribution of *CYP2C9*, *CYP2C19*, and *CYP3A4* alleles in Thai population. The figure was created using Microsoft Excel 365 (https://www.office.com).

The distribution of *CYP2C9*, *CYP2C19*, and *CYP3A4* alleles in different regions of Thailand are shown in Table 3 and Fig. 1. The frequency of the CYP2C9*3 allele was significantly lower among the Northern Thai population (P < 0.001). The frequency of the CYP2C19*17 allele was significantly higher in the Southern Thai population (P < 0.001). Overall, the prevalence of *CYP2C19* alleles was significantly different among the different regions of Thailand (P < 0.001). The frequency of the *CYP3A4* rs4646437 allele in different regions of Thailand was found to be comparable.

**Table 3:** Prevalence of *CYP2C9*, *CYP2C19*, and *CYP3A4* alleles in different regions of Thailand (n = 1205).

Allele	Minor allele frequency in different regions in Thailand					P-value (intragroup difference)	P-value (intergroup difference)
	BKK	Central	NE	Southern	Northern		
N	70	318	379	159	279		
CYP2C9*2	T = 0.000	T = 0.000	T = 0.001	T = 0.000	T = 0.002	0.564	0.832
CYP2C9*3	C = 0.057	C = 0.047	C = 0.049	C = 0.054	C = 0.010	< 0.001*	
CYP2C19*2	A = 0.229	A = 0.226	A = 0.294	A = 0.250	A = 0.253	0.916	< 0.001*
CYP2C19*3	A = 0.007	A = 0.025	A = 0.032	A = 0.028	A = 0.016	0.001	
CYP2C19*6	A = 0.000	A = 0.000	A = 0.001	A = 0.003	A = 0.000	0.317	
CYP2C19*17	T = 0.000	T = 0.020	T = 0.009	T = 0.051	T = 0.013	< 0.001*	
*CYP3A4* (rs4646437)	A = 0.192	A = 0.285	A = 0.299	A = 0.316	A = 0.265	0.199	

*Allele frequencies were compared in different regions of Thailand using χ2 test, P-value < 0.05.

*BKK* Bangkok, *NE* Northeastern.

### Frequencies of the *CYP2C9* and *CYP2C19* genotypes and predicted phenotypes in a sample of the Thai population

The *CYP2C9* and *CYP2C19* genotype and phenotype frequencies are summarized in Table 4. In our sample of the Thai population, 89.46% possessed a wild-type *CYP2C9* genotype while 10.54% possessed a mutated *CYP2C9* genotype. There were 1078 subjects (89.46%) classified as CYP2C9 NM (*1/*1), 125 subjects (10.37%) classified as CYP2C9 IM (*1/*2 and *1/*3), and 2 subjects (0.17%) classified as CYP2C9 PM (*3/*3).

**Table 4:** Frequencies of the *CYP2C9* and *CYP2C19* genotypes and predicted phenotypes in a sample of the Thai population (n = 1205).

Gene	Genotype	Genotype frequency, n (%)	Predicted phenotype	Predicted phenotype frequency, n (%)
*CYP2C9*	*1/*1	1078 (89.46)	NM	1078 (89.46)
	*1/*2	2 (0.17)	IM	125 (10.37)
	*1/*3	123 (10.20)		
	*3/*3	2 (0.17)	PM	2 (0.17)
*CYP2C19*	*1/*1	581 (48.22)	NM	581 (48.22)
	*1/*2	457 (37.93)	IM	518 (42.98)
	*1/*3	42 (3.49)		
	*1/*6	2 (0.16)		
	*2/*17	17 (1.41)		
	*2/*2	64 (5.31)	PM	80 (6.64)
	*2/*3	15 (1.24)		
	*3/*3	1 (0.08)		
	*1/*17	26 (2.16)	RM	26 (2.16)

*NM* normal metabolizer, *IM* intermediate metabolizer, *PM* poor metabolizer, *UM* rapid metabolizer.

Regarding *CYP2C19*, 48.22% possessed wild type *CYP2C19* genotype while 51.78% possessed a mutated *CYP2C19* genotype. There were 581 subjects (48.22%) classified as CYP2C19 NM (*1/*1), 518 subjects (42.98%) classified as CYP2C19 IM (*1/*2, *1/*3, *1/*6, and *2/*17), 80 subjects (6.64%) classified as CYP2C19 PM (*2/*2, *2/*3, and *3/*3), and 26 subjects (2.16%) classified as CYP2C19 RM (*1*17).

### Analysis of *CYP2C19* variants accordance between Luminex xTAG and TaqMan real-time PCR

We compared the allele and genotype frequencies pattern of *CYP2C19* variants between Luminex xTAG and TaqMan real-time PCR platforms. *CYP2C19* variants genotyped by TaqMan real-time PCR included *2, *3, and *17. *CYP2C19* variants genotyped by Luminex xTAG included *2, *3, *4, *5, *6, *7, *8, *9, *10, and *17. The presence of the wild type allele, CYP2C19*1, was inferred from the absence of other *CYP2C19* variants. Detailed comparisons of alleles and genotypes frequencies produced by the Luminex xTAG and TaqMan real-time PCR platforms are shown in Supplementary Tables 1 and 2. The accordant rate for allele and genotype frequencies was 98.89% (178/180) between the two platforms. The Kappa value was 0.931 indicating an almost perfect agreement between the two platforms.

## Discussion

The interethnic variability in the drug metabolism capacity and pharmacokinetics between populations is the impact of the differences in the allele distribution of pharmacogenes^14^. Drug metabolizing enzymes CYP2C9, CYP2C19, and CYP3A4 are polymorphic and cause substantial interpersonal differences in drug and metabolite exposure^15^. To date, numerous studies have analyzed the frequencies of the polymorphisms in *CYP2C9*, *CYP2C19*, and *CYP3A4* in the Thai population; yet the available frequency data have been derived from a small sample of the Thai population. In this study, we report the frequency of specific gene polymorphisms of *CYP2C9*, *CYP2C19*, and *CYP3A4* in 1,205 Thai subjects. Furthermore, this works are able to represent the majority allele frequencies of Thai people. Since the samples were recruited to cover all the regions in Thailand.

Regarding *CYP2C9*, the frequencies of the allelic variants CYP2C9*2 and CYP2C9*3 in the present study were consistent with the figures reported for East Asians in PharmGKB (https://www.pharmgkb.org/variant/PA166153972, accessed September 06, 2020). The frequency of CYP2C9*3 was slightly higher in our study compared to what has been reported in PharmGKB for East Asians (5.27% vs 3.37%). About differences in the frequency of alleles across different regions of Thailand, the frequency of the CYP2C9*3 allele was significantly lower among the Northern Thai population (Table 3). The CYP2C9*2 allele was observed with a frequency of 0.08% in our study. By contrast, the CYP2C9*2 allele frequency was zero in previous reports that were examined in Thais^16,17^. Pratt et al. reported that CYP2C9*2 and CYP2C9*3 have decreased function and are the most well-characterized variant *CYP2C9* alleles compared with other alleles^18^. Both CYP2C9*2 and CYP2C9*3 alleles have been reported at higher frequencies among the Caucasian population^19^. Concerning the metabolic predicted phenotypes, the frequencies of CYP2C9 IM and PM were 10.37% and 0.17%, respectively. Interestingly, these frequencies were more consistent with the Han Chinese population (8.23% IM and 0.16% PM)^20^. The Clinical Pharmacogenetics Implementation Consortium (CPIC) has published recommendations for pharmacogenetic testing on the *CYP2C9* gene for dosing of phenytoin, (NSAIDs), and warfarin^21–23^.

In our research, we determined and compared the frequency of four pharmacologically relevant *CYP2C19* variants among Thai populations. The frequencies of CYP2C19*2, CYP2C19*3, CYP2C19*6, and CYP2C19*17 were 25.6%, 2.5%, 0.1%, and 1.8% respectively. We observed the significant variability in the distribution of *CYP2C19* variants across different regions of Thailand. The CYP2C19*2, CYP2C19*3, and CYP2C19*6 alleles are the no-function alleles and characterized as PM, while the CYP2C19*17 allele is associated with high CYP2C19 activity and characterized as UM^24–27^. The frequency of the CYP2C19*2 allele in our study is less than the frequency in Asian populations (~ 30%) but higher than the allele frequency of 18% in Africans and Europeans^26^. The prevalence of the CYP2C19*3 allele presented in our study is lower than that of Han Chinese (7%), Koreans (12%), Japanese (11%), and Vietnamese (14%)^28^. The distribution of CYP2C19*2 and CYP2C19*3 alleles in Northeastern Thailand are consistent with the prior study of Tassaneeyakul et al. conducted in 774 Thais^29^. We observed a very low frequency of the CYP2C19*6 allele in our study. The prevalence of the CYP2C19*6 allele is very low, and sometimes absent in general populations^30^. The CYP2C19*17 allele, associated with accelerated metabolism, is prevalent at 18.2% in African-American, 6.2% in Asian, 15.8% in Caucasian, 15.2% in Hispanic, and 19.8% in Ashkenazi Jewish populations^30^. The frequency of the NM, IM, PM, and UM phenotypes was 48.22%, 42.98%, 6.64%, and 2.16% respectively. Notably, a similar trend was observed for the IM among the Thais and Han Chinese in previous studies^20,31^. The CYP2C19 metabolic phenotypes are uniformly distributed across African-American, Asian, Caucasian, Hispanic, and Ashkenazi Jewish populations^30^. Currently, there are dosing guidelines from the CPIC for *CYP2C19* genotyping and the personalization of clopidogrel, tricyclic antidepressants, selective serotonin reuptake inhibitors, voriconazole, and proton pump inhibitor therapy^32–36^.

The CYP3A4 enzyme is the most abundant metabolic enzyme in the human liver, encoded by the *CYP3A4* gene^37^. A lot of studies have reported the effects of *CYP3A4* rs4646437 on drug metabolism and outcomes of drug therapy^38–41^. The frequency of *CYP3A4* rs4646437 in our study was 28.5% in the Thai population and 29.9% in the northeastern Thai population, which is slightly higher from the findings in another study conducted in the northeastern Thai population^42^. According to the 1000 Genomes project, the *CYP3A4* rs4646437 is highly prevalent among African, East Asian, and South Asian populations, but not so frequent among the Europeans (https://www.pharmgkb.org/variant/PA166157391).

In addition to the allele, genotype, and phenotype frequencies in our study subjects, we also compared the Luminex xTAG v3 and TaqMan SNP genotyping methods for the detection of *CYP2C19* variants (Supplementary Table 3). Because of the lower turnaround time (~ 2.15 h), simple workflow, and lesser amount of DNA needed for PCR in TaqMan assay, we conclude that TaqMan SNP genotyping assay is better than Luminex xTAG v3 system for the detection of *CYP2C19* variants in Thai population.

## Conclusions

In conclusion, our findings confirm the interregional differences in the *CYP2C9*, *CYP2C19*, and *CYP3A4* allele and genotype frequencies among Thais. Our results further support the need to identify individuals who have altered pharmacokinetics for CYP2C9, CYP2C19, or CYP3A4 substrates so that prescribers could personalize appropriate dosages for optimal drug responses. TaqMan SNP genotyping assay is better than the Luminex xTAG v3 system in the genotyping of *CYP2C19* variants in the Thai population.

## Supplementary Information

Supplementary Tables.
