# Supplementary material for: Allele frequencies of single nucleotide polymorphisms of clinically important drug-metabolizing enzymes CYP2C9, CYP2C19, and CYP3A4 in a Thai population
Source: Sci Rep. 2021 Jun 11;11:12343. doi: 10.1038/s41598-021-90969-y (PMC8195986; doi:10.1038/s41598-021-90969-y)
Supplement: Supplementary file 1 — Supplementary Tables. [file 41598_2021_90969_MOESM1_ESM.docx]

**Allele frequencies of single nucleotide polymorphisms of clinically important drug-metabolizing enzymes *CYP2C9*, *CYP2C19*, and *CYP3A4* in a Thai population**

**Authors**: Rattanaporn Sukprasong^a,b^, Sumonrat Chuwongwattana^c^, Napatrupron Koomdee^a,b^, Thawinee Jantararoungtong^a,b^, Santirhat Prommas^a,b^, Pimonpan Jinda^a,b^, Jiratha Rachanakul^a,b^, Nutthan Nuntharadthanaphong^a,b^, Nutcha Jongjitsook^a,b^, Apichaya Puangpetch^a,b^, and Chonlaphat Sukasem^a,b^

**Supplementary Table 1. *CYP2C19* alleles and diplotypes frequencies by Taqman real-time PCR (n=180).**

| Allele | Frequency (%)  N (%) |
| --- | --- |
| **1* | 240 (66.67) |
| **2* | 102 (28.33) |
| **3* | 15 (4.17) |
| **17* | 3 (0.83) |
| Diplotype |  |
| *1/*1 | 80 (44.44) |
| *1/*2 | 69 (38.33) |
| *1/*3 | 10 (5.56) |
| *1/*17 | 2 (1.11) |
| *2/*2 | 14 (7.77) |
| *2/*3 | 3 (1.67) |
| *2/*17 | 1(0.56) |
| *3/*3 | 1(0.56) |

**Supplementary Table 2. *CYP2C19* alleles and diplotypes frequencies by Luminex xTAG (n=180).**

| Allele | Frequency (%)  N (%) |
| --- | --- |
| **1* | 239 (66.39) |
| **2* | 101 (28.05) |
| **3* | 15 (4.17) |
| **6* | 2 (0.56) |
| **17* | 3 (0.83) |
| Diplotype |  |
| *1/*1 | 79 (43.88) |
| *1/*2 | 68 (37.77) |
| *1/*3 | 10 (5.56) |
| *1/*6 | 1(0.56) |
| *1/*17 | 2 (1.11) |
| *2/*2 | 14 (7.77) |
| *2/*3 | 3 (1.67) |
| *2/*6 | 1(0.56) |
| *2/*17 | 1(0.56) |
| *3/*3 | 1(0.56) |

**Supplementary Table 3. Comparison between Luminex xTAG v3 and Taqman SNP Genotyping Assay for the detection of CYP2C19 variants.**

| Parameters | Luminex xTAG v3 | Taqman SNP Genotyping Assay |
| --- | --- | --- |
| Target Gene Capacity | *CYP2C19* (10 polymorphisms) | *CYP2C19* (3 polymorphisms) |
| Polymorphisms | 19154G>A), 17948G>A, 1A>G, C90033C>T, 12748G>A, 19294T>A, 12711T>C, 12784G>A, 19153C>T, -806C>T | G681G>A, 636G>A, -806C>T |
| The number of Variants,  Alleles detection of  *CYP2C19* gene | 10 alleles;  **2, *3, *4, *5, *6, *7, *8, *9, *10, *17* | 3 alleles;  **2, *3, *17* |
| Platform/ Detection Method | Bead-based array, Multiplex ASPE | TaqMan SNP genotyping with Real-time PCR |
| Turnaround time (TAT) | ~8.30 hours | ~2.15 hours |
| Process | 6 steps; 1. Extraction and Purification, 2. Multiplex PCR Reaction with Amplicon Treatment, 3. Allele Specific Primer Extension, 4. Bead Hybridization, 5. Addition of Reporter Molecule, 6. Data Acquisition and Analysis | 3 steps; 1. Denaturation, 2. Annealing, 3. Extension |
| Sample Type | Whole Blood EDTA or Citrate | Whole Blood EDTA |
| DNA Quantity (ng/PCR) | 4-300 ng/mL (24-1800 ng/PCR) | 1-20 ng genomic DNA/well |
| DNA Quality: A260/280 ratio | 1.5 | 1.8-2.0 |
| Number of samples per kit | 48 | 96-well plate |
| Instrument | Luminex 100/200 | Applied Biosystems ViiA7 system |
| Manufacturer | Luminex Molecular Diagnostics, Inc. | Applied Biosystems |
| FDA Cleared | May 2013 | Research Use Only |
